# Cytological analysis and structural quantification of FtsZ1-2 and FtsZ2-1 network characteristics in *Physcomitrella patens*

**DOI:** 10.1038/s41598-018-29284-y

**Published:** 2018-07-24

**Authors:** Bugra Özdemir, Pouyan Asgharzadeh, Annette I. Birkhold, Stefanie J. Mueller, Oliver Röhrle, Ralf Reski

**Affiliations:** 1 Plant Biotechnology, Faculty of Biology, University of Freiburg, Schaenzlestr. 1, 79104 Freiburg, Germany; 2 Institute of Applied Mechanics, University of Stuttgart, Pfaffenwaldring 7, 70569 Stuttgart, Germany; 3 INRES – Chemical Signalling, University of Bonn, Friedrich-Ebert-Allee 144, 53113 Bonn, Germany; 4 Stuttgart Center for Simulation Science (SimTech), University of Stuttgart, Pfaffenwaldring 5a, 70569 Stuttgart, Germany; 5 BIOSS – Centre for Biological Signalling Research, University of Freiburg, Schaenzlestr. 18, 79104 Freiburg, Germany; 6 Freiburg Center for Interactive Materials and Bioinspired Technologies (FIT), University of Freiburg, Georges-Köhler-Allee 105, 79110 Freiburg, Germany

## Abstract

Although the concept of the cytoskeleton as a cell-shape-determining scaffold is well established, it remains enigmatic how eukaryotic organelles adopt and maintain a specific morphology. The Filamentous Temperature Sensitive Z (FtsZ) protein family, an ancient tubulin, generates complex polymer networks, with striking similarity to the cytoskeleton, in the chloroplasts of the moss *Physcomitrella patens*. Certain members of this protein family are essential for structural integrity and shaping of chloroplasts, while others are not, illustrating the functional diversity within the FtsZ protein family. Here, we apply a combination of confocal laser scanning microscopy and a self-developed semi-automatic computational image analysis method for the quantitative characterisation and comparison of network morphologies and connectivity features for two selected, functionally dissimilar FtsZ isoforms, FtsZ1-2 and FtsZ2-1. We show that FtsZ1-2 and FtsZ2-1 networks are significantly different for 8 out of 25 structural descriptors. Therefore, our results demonstrate that different FtsZ isoforms are capable of generating polymer networks with distinctive morphological and connectivity features which might be linked to the functional differences between the two isoforms. To our knowledge, this is the first study to employ computational algorithms in the quantitative comparison of different classes of protein networks in living cells.

## Introduction

Eukaryotic cells are highly compartmentalized with entities that are each enclosed within a lipid bilayer. These compartments (organelles) normally have a stable shape, but can move and change their form dynamically^1,2^. Different proteins can aggregate to different types of filaments and build together the highly dynamic cytoskeleton that influences the shape and dynamics of cells and their organelles. It is far from understood how mechanical stability and shaping on the molecular scale function, because it is difficult to quantify, to analyse and to simulate the stability of this flexible system. One of the milestones in the field of cellular biomechanics was the concept of “cellular tensegrity”^3–5^, describing the biological cell as a tensegrity structure with cytoskeletal filaments representing both the tension and compression elements. This cellular tensegrity can help to understand various phenomena, such as the stability and dynamics of cell shape and the mechanotransduction^6,7^.

Chloroplasts are plant organelles with a diameter of a few micrometers surrounded by a lipid bilayer. They are, at least in land plants, usually lens-shaped. A few exceptions are described for mutant plants^8,9^. Proteins of the FtsZ (*filamentous temperature sensitive Z*) family are responsible for chloroplast division and shaping in *Physcomitrella patens*^10^, a moss and model plant^11^. This protein is homologous to tubulin, which is a part of the eukaryotic cytoskeleton, whereas in bacteria, FtsZ is part of the bacterial cytoskeleton. Here, it provides a scaffold for cell division. During establishment of endosymbiosis bacterial FtsZ genes were introduced by the bacterial ancestors of the plant organelles mitochondria and chloroplasts^12^. The presence of FtsZ proteins in *P. patens* was demonstrated and visualised *in vivo*^13,14^. The genome of *P. patens*^15–17^ encodes five FtsZ isoforms which group in three subclades (FtsZ1-1, FtsZ1-2, FtsZ2-1, FtsZ2-2 and FtsZ3), and thus more than in any other land plant studied so far^18,19^. The intracellular localisation of these five isoforms and their interaction were shown in this moss by fluorescence imaging and Förster resonance energy transfer (FRET) studies^13,20–22^. Their functions were revealed by reverse genetics via the targeted knockout of FtsZ genes facilitated by efficient homologous recombination^10^. All five moss FtsZ proteins have essential functions, some are necessary for plastid division. Based on differences in chloroplast sizes and shapes between the distinct loss-of-function mutants it can be concluded that during evolution a functional diversification of these proteins occurred^10^. FtsZ-GFP C-terminal fusions, which when slightly overexpressed enhanced chloroplast division in the moss, revealed complex patterns of FtsZ localisation and interaction^13,20^. Lu *et al*.^23^ suggested a double helix with a diameter of about 23 nanometer and two protofilaments as the molecular structure of bacterial FtsZ filaments.

The confocal laser scanning microscopy (CLSM) analysis of the FtsZ isoforms labelled with fluorescent reporters demonstrates that these proteins form complex filamentous networks in the chloroplasts of Physcomitrella. Moreover, as chloroplasts in loss-of-function mutants show distinct shape defects, these FtsZ networks might provide scaffolds that ensure the stability and structural integrity of the chloroplasts^24,25^. Thus, the term “plastoskeleton” (from “plastid” and “skeleton”) was coined for these FtsZ-based networks in moss plastids^24^. Overall, the plastoskeleton is reminiscent of geodesic domes, well-known in architecture.

Recent studies have found that moss chloroplasts are surrounded by a peptidoglycan wall, emphasising the evolutionary position of *P. patens* chloroplasts as intermediate between free-living bacteria and fully domesticated plastids of higher plants^25–27^. Because of this intermediate position and its experimental advantages, *P. patens* chloroplasts are suitable objects for live-cell visualization and subsequent modelling^28,29^.

As advanced microscopic imaging methods accumulate data on the morphological patterns of subcellular structures, the lack of computational methods for exploring the structural and functional details of subcellular structures becomes evident. The few existing three-dimensional computational models of these structures mainly focus on the cytoskeleton, cell morphogenesis or cell aggregates. These 3D computational representations are performed either by means of tensegrity networks^3–5^ or by continuum mechanical models which either simplify the structure to spatial trusses^30^ or use Finite Element analysis^31^. Many of these computational assessments are conducted on an isolated cell based on a single image data set or focus on extracting material parameters such as elasticity module based on such experimental techniques as Atomic Force Microscopy (AFM)^32–34^. A comprehensive analysis of these structures however, requires larger sample sizes in order to (i) take spatiotemporal variation (including cell-to-cell variation) into consideration, and consequently (ii) generalise/particularise the observed characteristics. This in turn requires development of automatic/semi-automatic computational methods capable of analysing large numbers of microscopic images. The difficulties associated with acquisition of appropriate image data and automatic reconstruction of computational models have prevented scientists from using 3D computational models to investigate structural and functional features of subcellular structures. Due to large amounts of data and inherent variation in the biological specimens, tailored steps in the image processing workflow e.g., fine tuning parameters of the adaptive thresholding algorithm, deconvolution and iterative processes for structural reconstruction are needed to encompass biological characteristics of FtsZ isoforms. The approach of transforming the intricate geometry of biological structures into a simpler representative form for the sake of computational analysis of these structures through a specifically developed process can be seen for example in Röhrle *et al*.^35^ where biological image data are utilised for extraction of the geometry for a finite element simulation. Furthermore, analysis of microscopic image data introduces challenges such as lower resolution in z-direction compared to in-plane resolution (x and y directions) and the greater effects of noise on the segmented image, which need to be taken into account.

FtsZ orthologs from various species are often studied with respect to their morphological, functional and dynamic assembly properties in the heterologous *S. pombe* (a yeast) expression system, providing valuable insights into the structural and functional properties of respective FtsZ orthologs^36–38^. In such studies, however, the comparative analysis of FtsZ network morphology between different isoforms, or under different experimental conditions is mainly based on visual judgement. We here perform 3D, semi-automatic quantitative analysis and comparison of FtsZ1-2 and FtsZ2-1 network morphology and connectivity in *P. patens* chloroplasts.

## Results

### No homologs of bacterial morphogenetic factors other than FtsZ are present in the *P. patens* proteome

We performed a homology search against the *P. patens* proteome version V3.3^17^ (cosmoss.org) to identify putative homologs of the bacterial cytoskeletal elements that were experimentally shown as important for bacterial morphogenetic processes. Some of these well-characterised bacterial morphogenetic factors include rod shape determinants MreB^39,40^, and RodZ^41^, curvature-inducing CreS^42^ and filamentous growth determinant FilP^43^. All these bacterial protein sequences used in the homology search were retrieved from the NCBI database^44^. The species and the strains that were used for obtaining protein sequences were selected based on experimentally proven morphogenetic function of the protein in those particular species and strains. The following species were selected for obtaining amino acid sequences of MreB: Nostoc sp. (PCC 7120)^45^, *Synechococcus elongatus* (PCC7942)^39^, *Caulobacter crescentus* (CB15)^40,46^, *Bacillus subtilis* (168)^47^, *Escherichia coli* (MG1655)^48^. For amino acid sequences of RodZ, *Escherichia coli* (MG1655)^41^ and *Escherichia coli* (BW25113)^41,49^ were selected. *Caulobacter crescentus* (CB15N)^42^ and *Streptomyces coelicolor* [A3(2)]^43^ were selected for the intermediate filament-like proteins CreS and FilP, respectively. These amino acid sequences were used in a homology search against the *P. patens in-silico* proteome at cosmoss.org using BLASTP^50^. All relevant information for these proteins including the NCBI accession numbers and the scientific publications in which the morphogenetic roles of the proteins are validated are summarized in Supplementary Table S1.

For the BLASTP analysis the substitution matrix BLOSUM62 with the threshold e-value 1e^−10^ was used. As a result, no homologs of these prokaryotic proteins were identified in *P. patens*. This finding indicates that most of the prokaryotic cytoskeletal factors involved in morphogenesis might have been lost during the evolution of plastids from cyanobacteria.

### FtsZ homologs show tissue- and growth-dependent expression patterns

According to the latest version of the *P. patens* genome^17^ this moss encodes five different FtsZ proteins which are grouped in three different subfamilies^19^: FtsZ1-1 (Pp3c22_4940.v3.1), FtsZ1-2 (Pp3c19_2490.v3.1), FtsZ2-1 (Pp3c11_17860.v3.1), FtsZ2-2 (Pp3c7_11570.v3.1) and FtsZ3 (Pp3c3_11140.v3.1). We analysed their gene expression *in silico* in all publicly available transcript data^51–53^, and found that the expression levels of *FtsZ* show dramatic differences both among the different members of the gene family, and across tissues and growth stages (Supplementary Fig. S1). According to these data, *FtsZ1-2* and *FtsZ2-1* show generally higher expression levels in the majority of the tested tissues than the remaining three isoforms. While *FtsZ2-2* shows a moderate expression in the majority of the tested tissues, *FtsZ1-1* and *FtsZ3* show the lowest expression of the whole gene family. Among the five different *FtsZ* genes, *FtsZ1-2* and the *FtsZ2-1* are particularly interesting because of their relatively higher expression levels in various tissues, a feature that could indicate the presence of a stable cytoskeleton-like scaffold, rather than the transient nature of a division ring, which would be required only during the division process. Therefore, we concentrated on these two isoforms.

### FtsZ1-2 and FtsZ2-1 knock-out mutants show different chloroplast phenotypes

We conducted a microscopic analysis involving the wild type (WT), the Δ*ftsZ1-2* and the Δ*ftsZ2-1* mutants to compare the respective chloroplast phenotypes in chloronema cells (Supplementary Fig. S1). We concentrated this analysis on chloronema cells, because this type of cell is the material from which protoplasts are obtained for transformation^9,54^ and because the subsequent cell type, caulonema cells, has a deviating chloroplast shape^11^. Chloroplasts of WT chloronema cells showed a roundish or elongated oval morphology. Some elongated chloroplasts also had slight indentations at the midpoint, indicating that they were in the process of division. The chloroplasts of Δ*ftsZ1-2* chloronema cells showed no morphological abnormalities compared to WT chloroplasts, suggesting that this isoform is not essential for maintaining the regular morphology of moss chloroplasts. In contrast, Δ*ftsZ2-1* chloronema cells exhibited severe defects in chloroplast shape and integrity, indicating an essential role for FtsZ2-1 in chloroplast morphogenesis (Supplementary Fig. S1). The dramatic differences in chloroplast phenotype in the chloronema cells of Δ*ftsZ1-2* and the Δ*ftsZ2-1* mutants cannot be explained by the differential expression of the two genes, since both show similar expression levels in the respective tissue (chloronema) (Supplementary Fig. S1).

### Analysis of the morphological and connectivity features of FtsZ1-2 and FtsZ2-1 networks

To achieve strong gene expression we created transient overexpression vectors, in which the coding sequences of FtsZ1-2 and FtsZ2-1 were fused at their C-terminus with the Enhanced Green Fluorescent Protein (EGFP). Bioreactor-grown WT *P. patens* protonema was used for the isolation of protoplasts^54^ which were subsequently transfected with these constructs. Confocal microscopy was conducted directly on live protoplasts between day 4 and day 7 when the signal intensity was optimal for image acquisition. The transfected protoplasts were extensively sampled via confocal microscopy. To improve the signal-to-noise ratio, a deconvolution process was applied to the entire pool of images. Lateral image resolution was about 140 nm after the deconvolution. For illustrations, maximum intensity projections, volume reconstructions and surface reconstructions were generated by using deconvolved z-stacks. For quantitative analysis, single chloroplasts were cropped and isolated from the other surrounding chloroplasts to facilitate the subsequent segmentation and image analysis. A sample size of 20 z-stacks for each isoform were digitally processed and set through specific quantitative structural analysis algorithms^55^ in order to decipher the characteristic network features for each isoform (please refer to Materials and Methods for further details).

### Visual analysis and comparison of FtsZ1-2 and FtsZ2-1 networks

**Figure 1:**
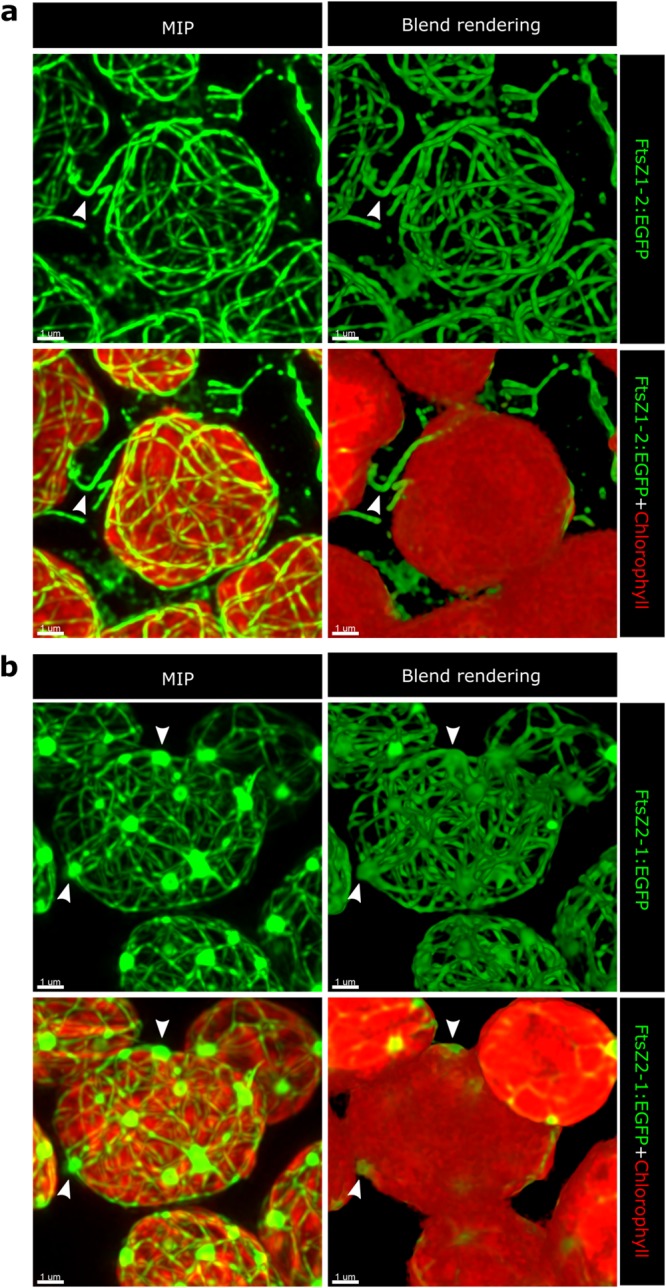
Examples of relatively complex networks and the corresponding chloroplasts hosting them are illustrated for each isoform. Confocal image z-stacks were deconvolved via Huygens software (Scientific Volume Imaging). For illustration, maximum intensity projection (MIP) and volume reconstruction (blend rendering) were applied to the deconvolved images via the IMARIS software (Bitplane). (**a**) FtsZ1-2 networks show uniform size for segments and nodes. A common feature of FtsZ1-2 networks is the presence of extraplastidic filaments which sometimes connect multiple chloroplasts of a cell (arrowheads). (**b**) FtsZ2-1 networks show detectable heterogeneity in node size with the meganodes being noticeably larger. The meganodes are usually located at the chloroplast surface (arrowheads). Unlike FtsZ1-2, in the networks of FtsZ2-1, no inter-plastidic connections are observed and occurrences of the extraplastidic filaments in general are very scarce.

**Figure 2:**
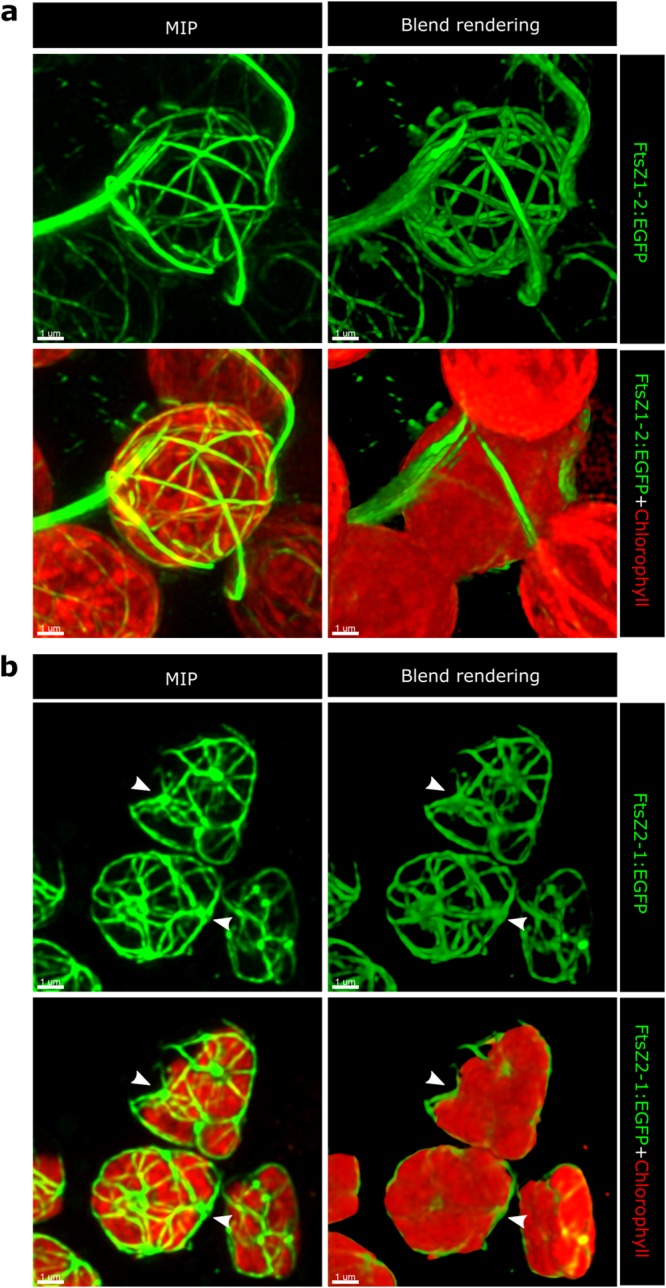
Examples of relatively simple networks and the corresponding chloroplasts hosting them are displayed. Confocal image z-stacks were deconvolved via Huygens software (Scientific Volume Imaging). For illustration, maximum intensity projection (MIP) and volume reconstruction (blend rendering) were applied to the deconvolved images via the IMARIS software (Bitplane). (**a**) FtsZ1-2 networks are characterised by extensive extraplastidic filaments. (**b**) FtsZ2-1 networks are interspersed with occasional meganodes (arrowheads). Visual analysis of the 3D images of the chloroplasts and FtsZ networks also reveals that the chloroplasts of the transfected cells often show morphological divergence from the standard chloroplast shape. This implies that elevated levels of FtsZ might induce morphological changes on the chloroplasts. Interestingly, the morphological deformation tends to have certain patterns which are isoform-specific.

Visual analysis of the FtsZ network images demonstrates that network structures of both isoforms vary in complexity, ranging from intricate (Fig. 1) to relatively simpler networks (Fig. 2). Furthermore, the networks of each isoform appear to display a broad diversity of geometric elements, including nodes and segments. Nodes are the intersections of the filaments in the networks and the segments are the pieces of filaments that connect one node to another. Both of these geometric elements show variable properties in both classes of networks. For instance, the nodes vary in size and number of connections they form and the segments may be straight or curved and show variability in length and thickness. Despite having these diverse geometric constitutions, networks of each specific isoform also possess unique features which can be considered geometric “hallmarks” of the individual network classes. A characteristic feature of the FtsZ1-2 networks is their ability to form long extraplastidic extensions emanating from the surface of the chloroplast and often connecting multiple chloroplasts of a cell (Fig. 1a, arrowheads). In contrast, the networks of FtsZ2-1 are largely confined within the volume of the chloroplast, forming no such interplastidic connections (Fig. 1b). FtsZ2-1 networks are interspersed with extraordinarily large nodes (henceforth referred to as meganodes) which are a characteristic feature of this isoform (Fig. 1b, arrowheads; Fig. 2b, arrowheads) and are not observed in the networks of FtsZ1-2. The meganodes behave like network hubs on which many filaments converge (Fig. 1b). It has to be noted that meganodes represent only a subset of the nodes in FtsZ2-1 networks, which is discussed further in the section “Quantitative structural analysis of FtsZ1-2 and FtsZ2-1 network nodes”.

**Figure 3:**
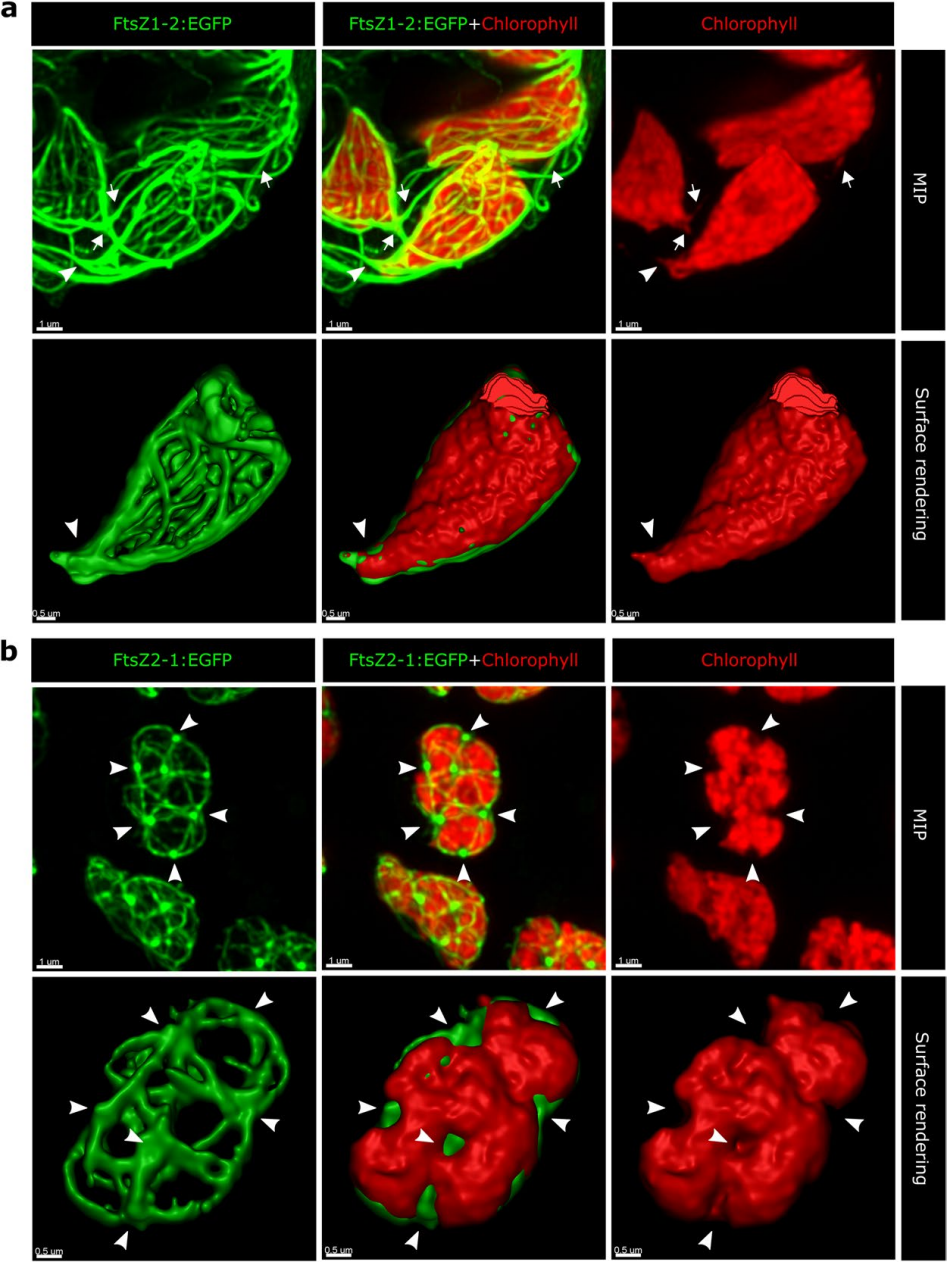
Morphological patterns of chloroplasts carrying FtsZ1-2::EGFP (**a**) and those carrying FtsZ2-1::EGFP (**b**) are shown for comparison. Confocal image z-stacks were deconvolved via Huygens software (Scientific Volume Imaging). Maximum intensity projections and surface rendering were performed on selected confocal image data for each isoform by using IMARIS software (Bitplane). Surface rendering was performed by choosing a single chloroplast in the middle of the image as the object and excluding the surrounding chloroplasts and networks during segmentation. (**a**) In the presence of FtsZ1-2::EGFP networks, chloroplasts tend to adopt angular shapes, form tapered and pointed poles where dense bundles of filaments exit the chloroplasts (arrowheads). FtsZ1-2 filaments emanating from the chloroplast surface are sometimes overlapped with chlorophyll autofluorescence in the form of tubular outgrowths (arrows) implying that the growing filaments might have deformed the chloroplast surface and triggered such membrane protrusions. (**b**) Shape patterns of chloroplasts carrying FtsZ2-1::EGFP include surface indentations which occur at positions where meganodes are located (arrowheads). Surface rendering of the chlorophyll and EGFP channels shows more clearly that locations of the meganodes match the topographic indentations of the chloroplast surface.

The chloroplasts with FtsZ1-2::EGFP often adopt angular shapes (Fig. 3a, Supplementary Video S1), show tapered chloroplast poles (Fig. 3a, arrowheads; Supplementary Fig. 2a, arrowheads; Supplementary Video S1) and, in more extreme cases, develop tubular extensions from the chloroplast surface (Fig. 3a, arrows; Supplementary Fig. 2a, arrows; Supplementary Video S1). These tubules are reminiscent of stromules, highly dynamic tubular extensions emanating from plastids^1,56–58^. However, unlike stromules, the tubules we observe show autofluorescence in the chlorophyll detection range, indicating that they contain chlorophyll. Intriguingly, these chlorophyll-containing tubules emanating from chloroplasts display co-alignment with FtsZ1-2 filaments (Fig. 3a, arrows; Supplementary Fig. 2a, arrows; Supplementary Video S1). In general, the chloroplasts carrying exogenous FtsZ1-2 protein demonstrate mostly outward-directed surface deformations, which are in the form of sharp angles, taperings and tubulations. In contrast, the chloroplasts carrying FtsZ2-1::EGFP show a different morphological pattern, consisting of surface indentations that are highlighted via surface rendering of the z-stacks (Fig. 3b, arrowheads; Supplementary Fig. 2b, arrowheads; Supplementary Video S2). On the chloroplast surface, the meganodes are located concomitantly with these indentations, hinting at a possible role of meganodes in the formation of such topographic variations (Fig. 3b, arrowheads; Supplementary Fig. 2b, arrowheads; Supplementary Video S2).

Altogether, the visual analysis of FtsZ1-2 and FtsZ2-1 networks and the chloroplasts hosting them reveals certain isoform-specific structural signatures in the networks and morphological deformations on the chloroplasts, which permit the discrimination between the two isoforms. Nevertheless, visual analysis is limited in terms of enabling an in-depth comparative analysis of the two classes of networks and identifying the significantly different morphological and connectivity features. To achieve these goals, the implementation of a quantitative and statistical approach is essential.

### Quantitative structural analysis of FtsZ1-2 and FtsZ2-1 network gross morphology

To carry out a quantitative comparison of structural features of FtsZ1-2 and FtsZ2-1 networks, we previously developed an image processing method for extracting structural characteristics of protein networks^55,59,60^. The extracted features quantitatively describe the structure of the networks from a global and a local perspective. The quantitative global portrayal of the network is performed by calculating enclosed volume, volume, density, greatest diameter, smallest diameter, stretch and oblateness of the network. Moreover, the local structural features of the network include number of nodes, node thickness, node density, node-to-node distance, node-to-surface distance, node-to-centre distance, node-to-surface to node-to-centre distance ratio, compactness, total number of segments, segment length, segment curvature, mean segment thickness, segment inhomogeneity, mean point-to-point distance, the mean number of connections per node, open nodes percentage, the mean angles between segments in 3 and 4 connection. From these 25 calculated descriptors, eight descriptors, i.e. enclosed volume of the network, network volume density, smallest diameter of the network, number of the nodes, node thickness, segment curvature, segment thickness and percentage of open nodes show significant difference for the FtsZ1-2 and FtsZ2-1 networks. Enclosed volume is the volume contained within the wrapped hull (the compartment bounded by the outermost surface of the gross network assembly). Network volume density is the ratio of the volume occupied by the actual network material to the enclosed volume. Smallest diameter of the network is the smallest diameter measured within the wrapped hull of a network. Number of nodes is the total number of nodes counted within a network. Node thickness is the average diameter calculated for a node. Segment curvature is the Menger curvature calculated for a segment. Segment thickness is the average diameter calculated along the length of a segment. Percentage of open nodes is the percentage within total number of nodes of those nodes which are connected to only one other node.

**Figure 4:**
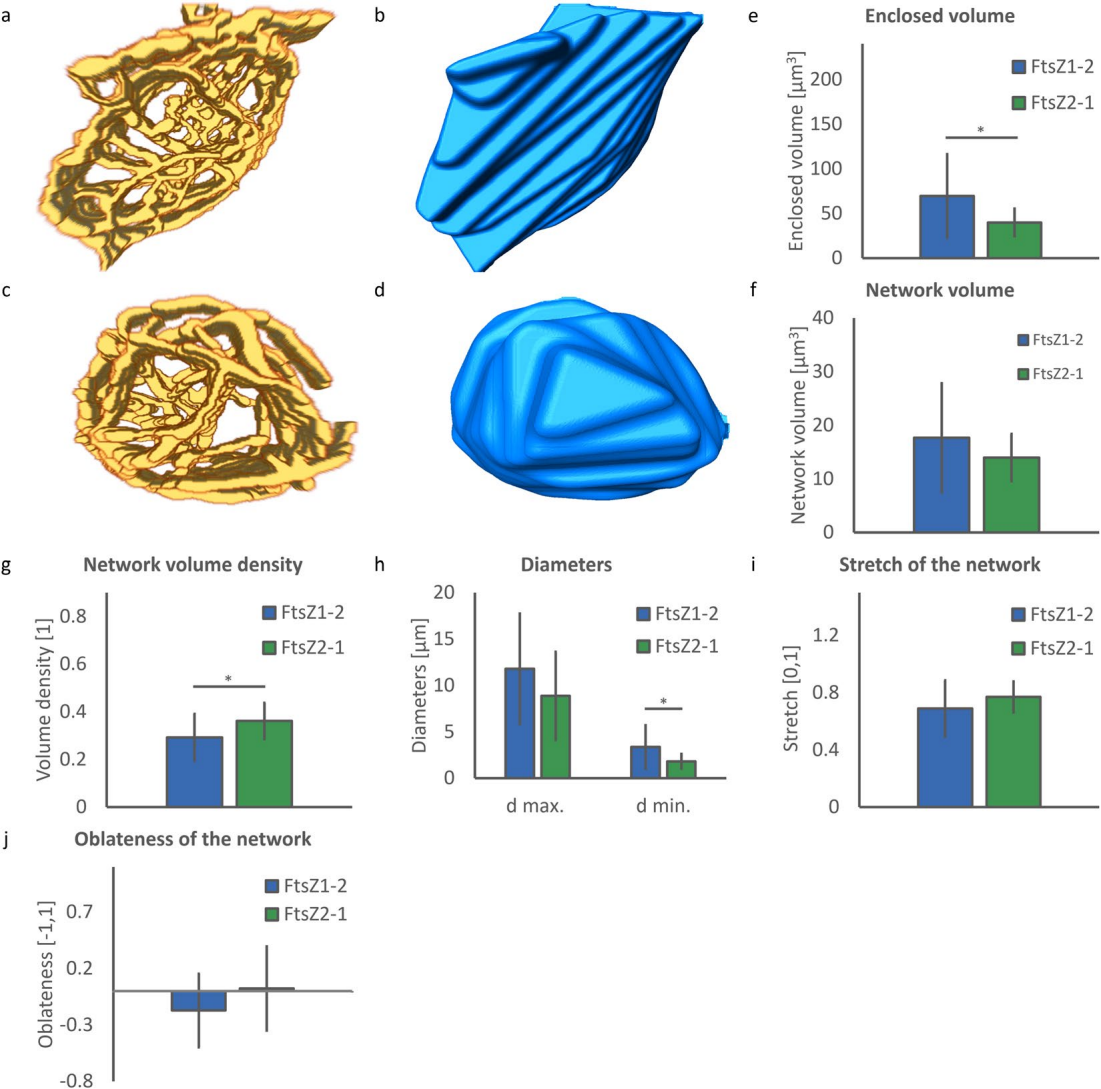
Analysis of gross morphological shape of FtsZ1-2 and FtsZ2-1 networks. (**a**) Representative sample of a segmented network of FtsZ1-2. (**b**) The outer surface (wrapped hull) of the segmented network in (**a**). (**c**) A representative sample of a segmented network of FtsZ2-1. (**d**) The outer surface created based on the wrapped hull of the segmented network in (**c**). (**e**–**j**) Quantitative analysis of network shape. A total of 40 networks (20 networks for each isoform) were used for each statistical analysis. Each network corresponds to a distinct chloroplast. FtsZ1-2 is shown in blue and FtsZ2-1 is shown in green colour. Data is shown as mean ± standard deviation. *Indicates significant difference between isoforms. (**e**) Enclosed volume of the network. (**f**) Network volume. (**g**) Network volume density. (**h**) Greatest and smallest diameters of the network. (**i**) Stretch of the network. (**j**) Oblateness of the network.

Before starting the analysis of the network gross morphology the deconvolved 3D confocal microscopy images were segmented using a semi-automated local adaptive threshold algorithm (Fig. 4a,c). The analysis of the overall shape of the networks was performed by calculating and analysing an outer surface surrounding the network micro-structures in the segmented images (Fig. 4b,d). Seven shape descriptors were calculated based on the combined analysis of the segmented network and this wrapped hull to quantitatively describe the overall morphology of the network.

Network shapes show a great range of variations. However, quantitative comparison of the network structures belonging to the two isoforms reveals that three out of seven structural features (enclosed volume, network volume density and small diameter of the network) are distinctive (statistically significantly different) features, while the four other shape descriptors are generic. The volume enclosed by FtsZ1-2 networks (69.5 ± 48.9 µm^3^) is significantly greater than that enclosed by FtsZ2-1 (39.9 ± 15.4 µm^3^) networks (p = 0.01, Fig. 4e). However, FtsZ1-2 and FtsZ2-1 networks have similar network volumes (17.7 ± 10.2 µm^3^ and 14.0 ± 4.4 µm^3^, respectively, Fig. 4f). This reveals that space not occupied by material within the FtsZ1-2 networks is greater than in FtsZ2-1. Quantitatively, this can be seen in the significantly lower network volume density for FtsZ1-2 (0.30 ± 0.10) compared to that for FtsZ2-1 (0.36 ± 0.07, p = 0.02, Fig. 4g). Besides volume and density, the overall shapes of the networks show differences between the two isoforms, as the smaller diameter in FtsZ1-2 is significantly greater than the one in FtsZ2-1 (3.34 ± 2.36 µm vs. 1.80 ± 0.79 µm, p = 0.01, Fig. 4h). In contrast, the greatest diameter of the networks (FtsZ1-2: 11.8 ± 5.96 µm and FtsZ2-1: 8.86 ± 4.76 µm), the stretch of the networks (FtsZ1-2: 0.69 ± 0.20 and FtsZ2-1: 0.77 ± 0.11) and the oblateness of the networks (FtsZ1-2: −0.17 ± 0.35 and FtsZ2-1: 0.02 ± 0.37) are not significantly different for the two isoforms (Fig. 4h–j). However, the different signs for the oblateness value for FtsZ1-2 (negative) and FtsZ2-1 (positive) point towards a more flat and plate-like shape of FtsZ1-2 networks.

### Quantitative structural analysis of FtsZ1-2 and FtsZ2-1 network nodes

**Figure 5:**
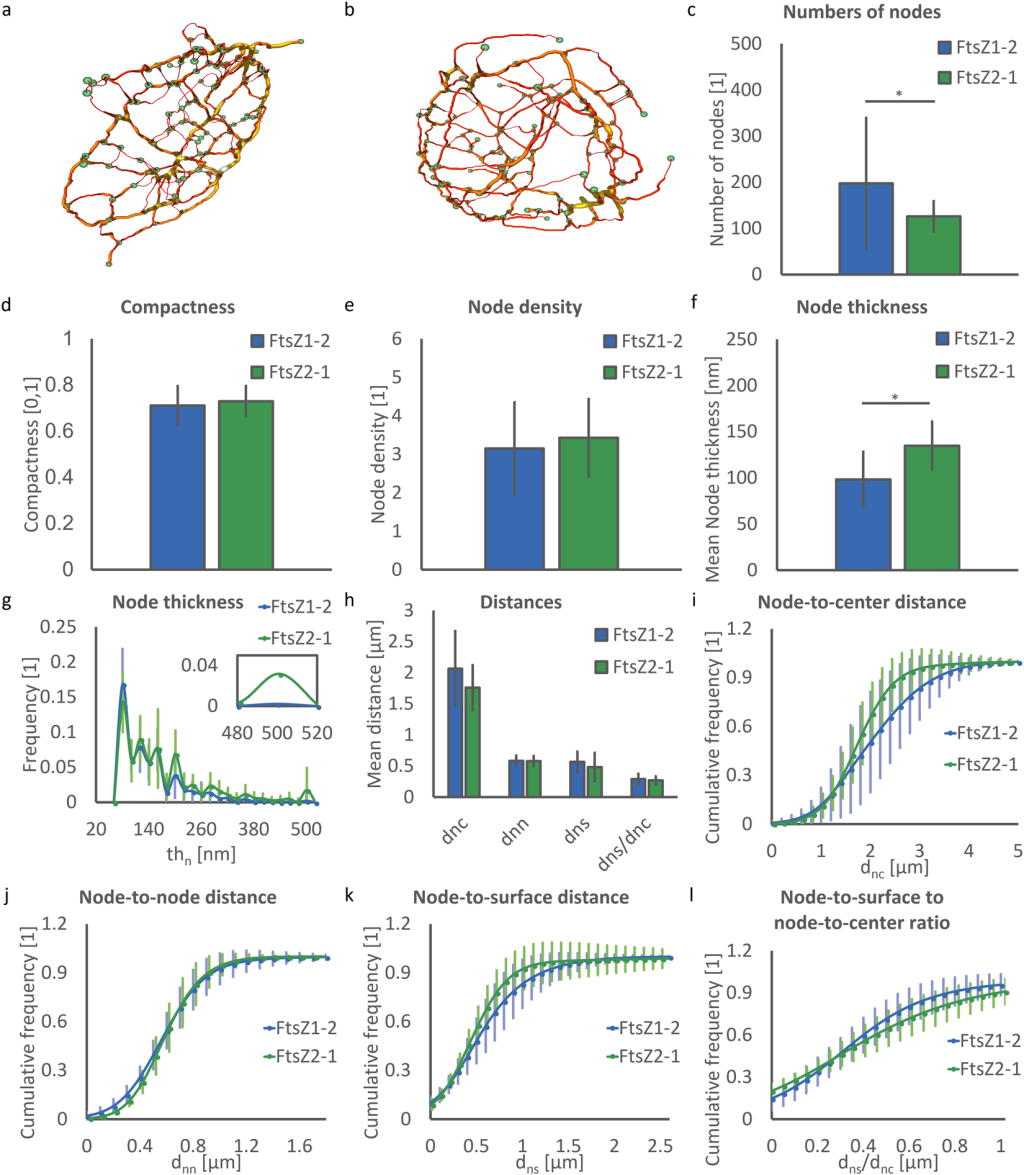
Spatial graphs and evaluated nodal parameters of FtsZ1-2 and FtsZ2-1. (**a**) A sample spatial graph of a FtsZ1-2 network. (**b**) A sample spatial graph of a FtsZ2-1 network. Nodes in both networks of (**a**) and (**b**) are shown in green spheres. The segments are colour coded based on their thickness with red (thinner segments) → yellow (thicker segments). (**c**) Number of nodes. (**d**) Compactness of the network. (**e**) Node density. (**f**) Mean values of node thickness per network. (**g**) Normalized distribution of node thickness. Inset shows the sizes of meganodes. (**h**) Mean values of node-to-centre distance per network, node-to-node distance, node-to-surface distance and node-to-surface to node-to-centre distance ratio. (**i**) Normalized cumulative distribution of node-to-centre distance. (**j**) Normalized cumulative distribution of node-to-node distance. (**k**) Normalized cumulative distribution of node-to-surface distance. (**l**) Normalized cumulative distribution of node-to-surface to node-to-centre distance ratio. (**c**–**l**) A total of 40 networks (20 networks for each isoform) were used for each statistical analysis. Each network corresponds to a distinct chloroplast. For each plot FtsZ1-2 and FtsZ2-1 are represented in blue and green, respectively, and data is shown as mean ± standard deviation. In the bar plots * indicates significant difference between isoforms. (**c**–**e**) One value per network. (**f**,**h**) Mean of all nodes per network. (**g**) Normalized distribution of all nodes per network. (**i**–**l**) Normalized cumulative distribution of all nodes per network. (**g**–**l**) n = 3947 and 2526 nodes for FtsZ1-2 and FtsZ2-1 analysed, respectively.

To analyse morphological details of the structural components of the protein network, the segmented images were transformed into a spatial graph consisting of points, nodes, segments and connections (Fig. 5a,b). Points were placed at filaments where a local change in segment characteristic such as orientation or thickness occurs. Elements are the structural component connection points. Nodes are defined as points that are connected to more than two other points. Nodes are meeting points of the filaments in the network. Segments are the filaments connecting one node to another. For a quantitative characterisation of these elements, structural features were computed.

FtsZ1-2 networks consist of significantly more nodes than FtsZ2-1 networks (197 ± 142 vs. 126 ± 32, p = 0.04, Fig. 5c). The number of nodes shows a great distribution within the class of FtsZ1-2 networks. Despite the difference in the number of nodes in the networks, compactness and node densities, which are directly related to the number of nodes, are not statistically different for the two isoforms (FtsZ1-2: 0.71 ± 0.09 and 3.15 ± 1.20, respectively and FtsZ2-1: 0.72 ± 0.07 and 3.43 ± 1.00, respectively; Fig. 5d,e). This is due to the similarities in network volume being the dominant parameter in the evaluation of these two shape descriptors. The mean node thickness is another distinctive feature, showing a significant difference between the FtsZ1-2 and FtsZ2-1 isoforms (mean value per network: FtsZ1-2: 98.3 ± 29.9 nm and FtsZ2-1: 134.8 ± 26.2 nm (p = 0.00), respectively, Fig. 5f). Moreover, the analysis of the normalised distributions of all node thicknesses in the networks reveals noteworthy results (Fig. 5g). For nodes with thickness up to 480 nm the distributions for the two isoforms are very similar. However, in FtsZ2-1 networks a small portion of nodes (approximately 3%) have thickness values between 480–520 nm whereas in FtsZ1-2 networks no nodes of these sizes exist. This small fraction of nodes in FtsZ2-1 networks comprises the meganodes. The analysis of relative distances reveals that in both classes of networks individual nodes are located closely to the surface and other nodes, but are further away from the centre of the network. In FtsZ1-2 networks there is a trend of this distance to the centre being even greater than in FtsZ2-1 (p = 0.06). 78% of all nodes of the FtsZ2-1 networks lie within a distance of less than 2.5 µm from the centre of gravity of the network, whereas in FtsZ1-2 networks only 59% are within the same distance (Fig. 5i). On the other hand, more nodes in FtsZ2-1 networks are located closely to the network surface (90% vs. 80% within 1 µm, Fig. 5k). Besides this difference, local node distributions in the networks are similar (Fig. 5j,l). Mean node-to-centre distances (FtsZ1-2: 2.07 ± 0.60 µm and FtsZ2-1: 1.76 ± 0.36 µm), node-to-node distances (FtsZ1-2: 0.58 ± 0.08 µm and FtsZ2-1: 0.59 ± 0.08 µm), node-to-surface distances (FtsZ1-2: 0.57 ± 0.16 µm and FtsZ2-1: 0.48 ± 0.23 µm) and node-to-surface to node-to-centre distance ratio (FtsZ1-2: 0.29 ± 0.09 and FtsZ2-1: 0.27 ± 0.07, Fig. 5h) are not statistically different between the two isoforms.

### Quantitative structural analysis of FtsZ1-2 and FtsZ2-1 network segments

**Figure 6:**
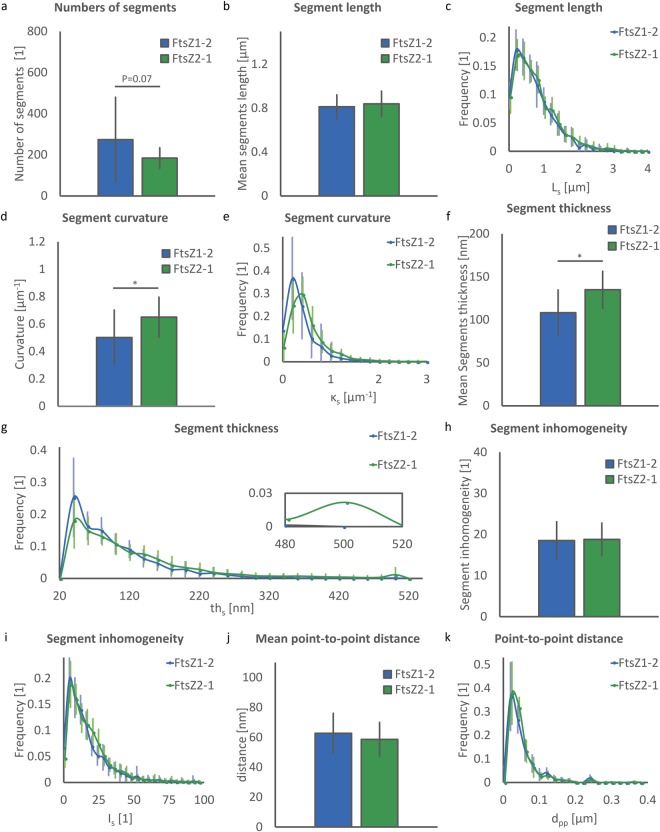
Evaluation of element morphology. (**a**) Number of segments. (**b**) Mean values of segment length. (**c**) Normalised distribution of segment length. (**d**) Mean values of segment curvature per network. (**e**) Normalised distribution of segment curvature in FtsZ1-2 and FtsZ2-1 isoforms. (**f**) Mean values of segment thickness. (**g**) Normalised distribution of segment thickness. Inset shows segments above 480 nm thickness. (**h**) Segment inhomogeneity. (**i**) Normalised distribution of segment inhomogeneity. (**j**) Mean values of point-to-point distance per network. (**k**) Normalised distribution of point-to-point distance. (**a**–**k**) A total of 40 networks (20 networks for each isoform) were used for each statistical analysis. Each network corresponds to a distinct chloroplast. For each plot FtsZ1-2 and FtsZ2-1 are represented in blue and green, respectively and data is shown as mean ± standard deviation. In the bar plots * indicates significant difference between isoforms. (**a**) One value per network. (**b**,**d**,**f**,**h**) mean of all segments per network. (**c**,**e**,**g**,**i**,**k**) normalised distribution of all segments per network. (**a**–**k**) 5472 and 3676 segments were analysed for FtsZ1-2 and FtsZ2-1 respectively.

The number of segments in FtsZ1-2 (237 ± 206) shows a great variation and is slightly, but not significantly greater than in FtsZ2-1 (183 ± 49.4, p = 0.07, Fig. 6a). In contrast, segment length (mean length: FtsZ1-2: 0.81 ± 0.11 µm and FtsZ2-1: 0.84 ± 0.12 µm, Fig. 6b) and its normalised distribution (Fig. 6c) are remarkably similar in both networks. The segments in FtsZ1-2 networks are significantly less curved than in FtsZ2-1 (0.50 ± 0.20 µm^−1^ and 0.65 ± 0.14 µm^−1^, respectively, Fig. 6d) resulting in another distinctive element descriptor (p = 0.01). Despite the significant difference in the mean values, distributions of curvatures in the two classes of networks are similar (Fig. 6e). Analogously, the segments in FtZ2-1 networks are on the average thicker than the ones in FtsZ1-2 networks (134.9 ± 21.4 nm and 108.2 ± 26.5 nm, respectively; p = 0.00, Fig. 6f). Although the normalised distributions of segment thickness (Fig. 6g) for both isoforms below a thickness of 480 nm are very similar, almost 4% of the segments in FtsZ2-1 networks have a thickness between 480 and 520 nm while almost no segments in FtsZ1-2 network show such high thickness values. These thick segments in FtsZ2-1 are the ones meeting at the meganodes. The mean and normalised distributions of segment inhomogeneity (Fig. 6h,i) are almost identical in both isoforms, (FtsZ1-2: 18.5 ± 4.56 and FtsZ2-1: 18.8 ± 3.97). The same pattern is observed for point-to-point distances (FtsZ1-2: 62.7 ± 13.3 nm and FtsZ2-1: 58.7 ± 11.4 nm, Fig. 6j) and its normalised distribution (Fig. 6k).

### Quantitative structural analysis of FtsZ1-2 and FtsZ2-1 network connections

**Figure 7:**
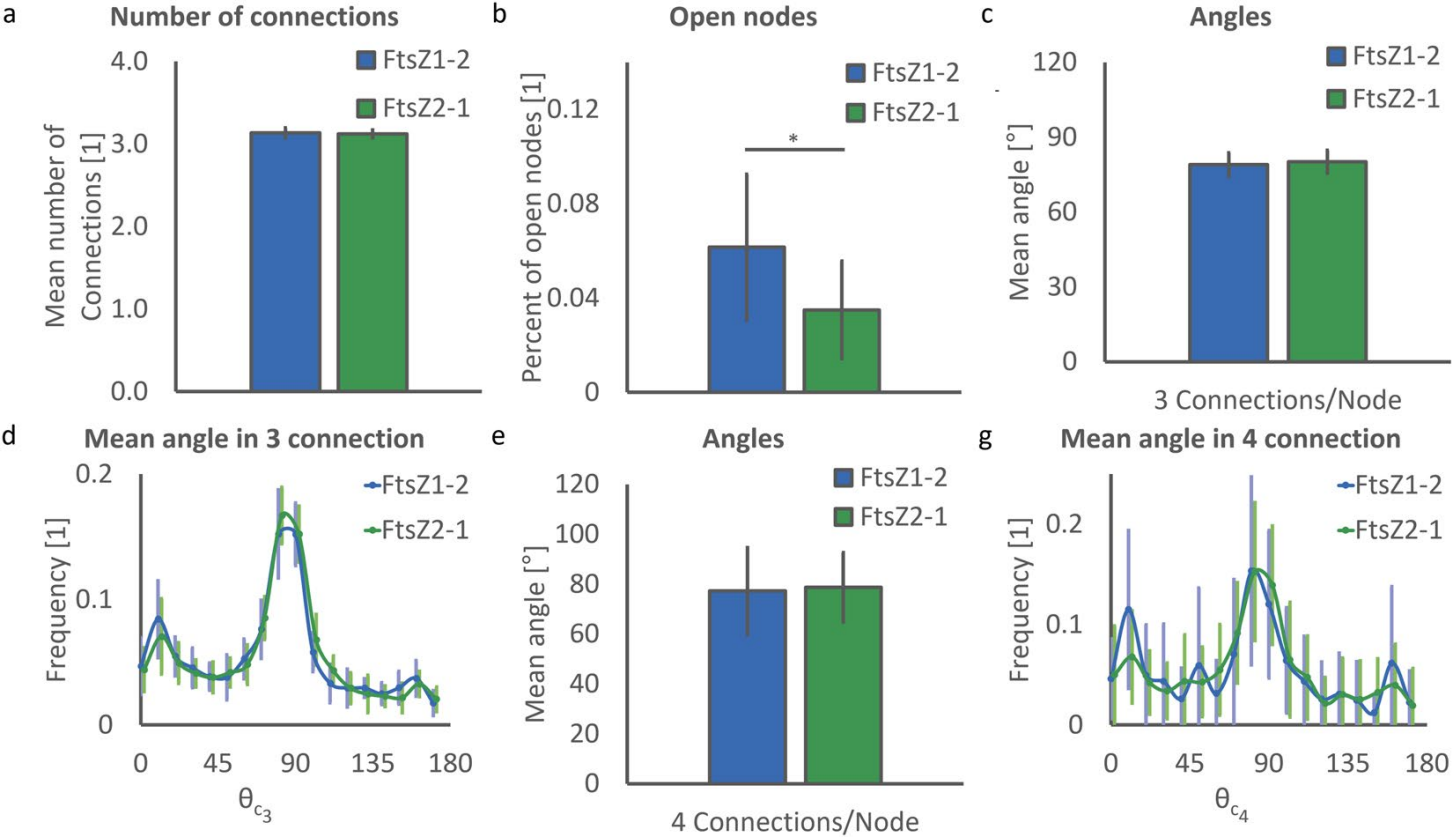
Evaluation of connections. (**a**) Mean number of connections per network. n = 12468 and 7905 connections for FtsZ1-2 and FtsZ2-1, respectively. (**b**) Percentage of open nodes. n = 237 and 87 open nodes for FtsZ1-2 and FtsZ2-1, respectively. (**c**) Mean values of angles between segments in nodes with three connections. (**d**) Normalized distribution of mean angles in nodes with three connections. (**e**) Mean values of angles between segments in nodes with four connections. (**g**) Normalized distribution of mean angles in nodes with four connections. (**c**–**g**) n=6648 and 5163 for 3-per-node connections and 966 and 691 for 4-per-node connections for FtsZ1-2 and FtsZ2-1, respectively. (**a**–**g**) A total of 40 networks (20 networks for each isoform) were used for each statistical analysis. Each network corresponds to a distinct chloroplast. For each plot FtsZ1-2 and FtsZ2-1 are represented in blue and green, respectively and data is shown as mean ± standard deviation. In the bar plots *indicates significant difference between isoforms.

The number of connections in the two isoforms are very similar (FtsZ1-2: 3.14 ± 0.06 and FtsZ2-1: 3.12 ± 0.05, Fig. 7a). This similarity is due to the fact that both networks contain nodes with mostly three connections. On the contrary, the percentage of open nodes is significantly higher in FtsZ1-2 networks (FtsZ1-2: 6.14 ± 3.10% and FtsZ2-1: 3.50 ± 2.06%, p < 0.01; Fig. 7b). This demonstrates the tendency of the filaments in FtsZ to leave the chloroplast which is evaluated as an open node in our quantification method. The low p value in this analysis is also consistent with the fact that extraplastidic filaments are much more frequently observed in FtsZ1-2 networks than in FtsZ2-1 networks. Assessment of the mean values and distribution of angles between segments in the most observed connections (three and four connections, Fig. 7c–g, respectively) reveals similar connectivity inside FtsZ1-2 and FtsZ2-1 networks. Both networks have angles around 80° in nodes with three and four connections (FtsZ1-2: 78.9 ± 4.78° and 77.2 ± 17.4° and FtsZ2-1: 80.1 ± 4.60° and 78.7 ± 13.9°, respectively). It is worth noting that in both network types the standard deviation of angles in nodes with four connections is higher than in nodes with three connections (Fig. 7g).

## Discussion

The role of FtsZ in cellular morphogenesis has been studied mainly in prokaryotes. In such studies, certain mutant forms of this protein were linked to defective shaping of bacterial cells^61–63^. Such cell shape defects might result from defective septal peptidoglycan synthesis, a process controlled by FtsZ treadmilling^64–67^. Nevertheless, other studies demonstrate that FtsZ is not only localised at the septum but also uniformly spread throughout the cytoplasm in cyanobacteria^68^. FtsZ can create spatial motifs other than rings, such as the helix and spiral-like patterns in *Bacillus subtilis*^69^, *Escherichia coli*^70^, *Streptomyces coelicolor*^71^ and *Helicobacter pylori*^72^. In addition to prokaryotic cells, such spiral-like forms of FtsZ also exist in chloroplasts of *Arabidopsis thaliana*^73–75^. In general, such studies indicate that FtsZ is capable of adopting shapes other than rings and can be localised to sites different from the mid-cell.

In plants FtsZ proteins are encoded by nuclear genes, translated in the cytosol, and imported into chloroplasts. FtsZ proteins share similarities with the cytoskletetal tubulin proteins and are important for chloroplast division^10,14^. In addition, overexpressed FtsZ proteins have been shown to form filamentous networks in the chloroplasts of *A. thaliana*^73^ and *P. patens*^21^.

*P. patens* encodes five different FtsZ isoforms^17,76^, which so far is the highest recorded number for any species^18,19^. Based on similarities between the cytoskeleton and the FtsZ filaments inside chloroplasts, Reski^24^ proposed the term “plastoskeleton” for these networks. Some FtsZ isoforms occur also in the cytosol^21,22^ and they can specifically interact *in vivo* in both cellular compartments^20^, which together may explain why these five proteins have overlapping functions in plastid division, shaping of the organelle, cell patterning, development of the whole moss plant, and sensing of gravity^10^. Indeed, despite the presence of five different isoforms of the tubulin homolog FtsZ we could find no homologs of bacterial actin-homologs such as MreB or bacterial IFs such as CreS in the *P. patens* genome. Therefore, it could be possible that through functional diversification, the FtsZ family might have given rise to all functional elements needed for a shape-determining scaffold in chloroplasts. Among the five different single-gene knockout lines in moss, the Δ*ftsZ1-2* and the Δ*ftsZ2-1* are particularly interesting with respect to their chloroplast phenotype. While the genes *FtsZ1-2* and *FtsZ2-1* show similar levels of expression in the wild type chloronema cells, the chloroplast morphology shows a drastic difference in the same tissue of the knockout lines for the respective genes. This implies that the morphological differences between the chloroplasts of the two mutant lines are likely due to the differences in sequence, structure and function of the two isoforms. As the sequence, structure and function of the monomeric isoforms could strongly influence the 3D architecture of the networks formed by them, the differences between the isoforms regarding those aspects would be reflected at the scale of polymer networks formed by the two isoforms. Therefore, a comprehensive analysis of the network morphology and connectivity for each FtsZ isoform and identification of the distinctive network features between the different isoforms could provide hints at the specific functions of these proteins. In particular, biomechanical analyses of relationships between the FtsZ networks and the chloroplast shape and integrity would benefit from such a comparative morphological characterisation. Because the concept of a plastoskeleton is still a matter of debate^1,25,77,78^ we approach this question with a combination of cytological characterisation and quantitative image analysis^55,59,60^. As an entry into the computer modelling and simulation of protein networks, we describe here the comparative structural analysis of the FtsZ1-2 and FtsZ2-1 network morphology and connectivity.

A visual inspection of the 3D-reconstructed images of FtsZ1-2 and FtsZ2-1 networks reveals only few distinctive features between the two isoforms. These include the extraplastidic filaments of FtsZ1-2 networks and the meganodes of FtsZ2-1 networks (Figs 1 and 2). Both of these observations, which are based on visual inspection, are confirmed by the quantitative analysis which reveals that i) the proportion of the open nodes in FtsZ1-2 networks is significantly higher than that in FtsZ2-1 networks (Fig. 7b) and that ii) the meganodes occur only in the FtsZ2-1 networks where they constitute about 3% of all nodes (Fig. 5g). Therefore, our method of quantitative analysis proves to be useful in testing the validity of visually detected structural phenomena and also quantifies the extent to which these phenomena are represented in the respective network classes.

Besides testing the validity of already detected distinctions, our quantitative analysis also identifies further distinctive features which avoid detection via visual inspection of images. These distinctive features include i) shape descriptors (Fig. 4) that provide information about the global morphology of the networks and ii) elemental descriptors (Figs 5, 6 and 7) that characterise the smallest-scale components of the network assembly. A combined analysis of the shape descriptors and elemental descriptors reveals interesting outcomes. For instance, the volume occupied by the network material (network volume, Fig. 4f) is generic between the two isoforms although the number of nodes is significantly lower for FtsZ2-1 networks (Fig. 5c) and the number of segments also tends to be lower for the networks of this isoform (Fig. 6a, p = 0.07). Some of the other descriptors related to the network volume are node thickness (Fig. 5f) and segment length (Fig. 6b), where node thickness shows a significant difference between the two isoforms while segment length calculations are similar between networks. Moreover, node thickness (Fig. 5f) and segment thickness (Fig. 6f) are the only material quantity descriptors which are greater for FtsZ2-1 networks and which obviously compensate for the lower node and segment number in the FtsZ2-1 network volume, leading to a generic network volume for both isoforms. As FstZ1-2 and FtsZ2-1 monomers are close to each other in the length of their amino acid sequence, and thus are likely to have a similar size in the monomeric structure, the greater thickness of the FtsZ2-1 (Fig. 6f) filaments points towards a greater tendency of FtsZ2-1 filaments to form bundles compared to the filaments of FtsZ1-2. Morphological features of protein filaments have long been studied with the goal of understanding the load-bearing properties of cytoskeletal components such as microfilaments and microtubules^3,6^. In such studies, the actin networks, having straight filaments *in vivo* are suggested to be in tensile stress, whereas microtubules are often found to be curved *in vivo* and are thus considered to be buckled as they oppose compressive load^3,6^. In addition to having more curved morphology than microfilaments the microtubules also show greater thickness, a feature that favours their putative role as stiff load-bearing elements. Interestingly, our quantitative comparison identifies FtsZ2-1 filaments to be thicker (Fig. 6f) and more curved (Fig. 6d) *in vivo* than FtsZ1-2 filaments. Regarding both morphological features they are more similar than FtsZ1-2 filaments to the microtubules of the cytoskeleton. This morphological similarity might be indicative of greater compressive load-bearing properties for the filaments of FtsZ2-1 compared to those of FtsZ1-2.

It is important to note that the evaluated values for the small-sized features such as node and segment thicknesses might be affected by the limited resolution of confocal microscopy. Furthermore, some of the determined values might also depend on the image processing algorithms, which are used for the quantification of the structures. Therefore, especially for small-sized features such as node and segment thicknesses the calculated values have to be interpreted carefully, when deriving functional conclusions.

In addition to such structural features that are significantly different between the two isoforms, the quantitative analysis also provides information about the general behaviour and trends that the two network classes show. For instance, nodes of the networks are not evenly distributed within the enclosed network volume. Rather, they are located relatively closely to the surface of the networks and far away from the network centre (Fig. 5h,i,k,l). This proximity to the surface could be due to a possible function that the nodes may have at the chloroplast surface. This result is also consistent with the findings of the visual analysis that meganodes of FtsZ2-1 are often observed at the chloroplast outer surface and associated with surface indentations (Fig. 3b, Supplementary Fig. S2b, Video S2).

Comparisons of the FtsZ networks and the chloroplasts containing them reveal that certain isoform-specific network features occur at the sites of specific structural deformation patterns at the chloroplast surface (Fig. 3, Supplementary Fig. 2, Videos S1 and S2). These altered chloroplast morphologies detected in the transfected cells deviate from the regular shape of the chloroplasts normally observed in the non-transfected cells, and could thus be attributed to the elevated levels of the respective FtsZ protein.

Morphological deformations of the chloroplasts carrying exogenous FtsZ1-2 include sharp corners, tapered poles and tubulations from the surface (Fig. 3a, Supplementary Fig. 2a, Video S1), all of which imply outward-directed forces being exerted on the chloroplast surface. The general patterns of FtsZ1-2 filaments, extending out of chloroplasts and connecting multiple chloroplasts are reminiscent of stromules – stroma-filled membranous tubules that emanate from plastid surface. Stromules may play roles in plant innate immunity, inter-organellar communication and exchange of molecules, plastid division and shaping and intracellular positioning and orientation of plastids^1,79–83^. Therefore, our observation of FtsZ1-2 filaments in the form of interplastidic connections suggests a possible localisation of this isoform in stromules and hence a function for this FtsZ isoform in stromule-related biological processes. Concerning the mechanical basis of stromule formation various hypotheses were proposed, including a role of external pull forces by the cytoskeleton^80,81^. Our data now suggest that FtsZ1-2 filaments may have a role as an internal source of force in the formation of stromules.

Chloroplasts with exogenous FtsZ2-1 networks demonstrate inward-directed indentations at the chloroplast surface (Fig. 3b, Supplementary Fig. 2b, Video S2). Interestingly, the meganodes of FtsZ2-1 are located at these surface indentations, an observation that suggests that the indentations could be triggered by the meganodes. In this scenario meganodes would represent contact points where the FtsZ2-1 networks are anchored to the chloroplast envelope. A possible uneven growth of the FtsZ2-1 networks, where the network grows slower at the sites of the meganodes, the chloroplast growth would also be opposed at these sites due to the attachment of the meganodes to the envelope, eventually leading to the indented surfaces we observe.

In this study, we show that computational algorithms can be useful for extracting quantitative features of protein network morphology and connectivity. This is also the first study to perform a quantitative comparison of network features between two different protein isoforms in live cells.

### Outlook

In order to gain a mechanistic understanding of FtsZ function in chloroplast morphogenesis, the early-to-late progression of network formation and relevant changes in chloroplast morphology can be monitored via time-lapse fluorescent imaging. Our quantitative image analysis method can be adapted for detection of the temporal changes in the network organisation and turnover of FtsZ filaments. To learn about the mechanical properties of FtsZ networks, laser ablation microscopy could be implemented; this would pave the path to a realistic simulation of the plastoskeleton.

Recent studies have also demonstrated that chloroplasts of some eukaryotic organisms, including green algae and mosses, are surrounded by a peptidoglycan wall^27,84^. Investigation of interactions between various peptidoglycan biosynthetic enzymes and FtsZ isoforms could reveal further roles for FtsZ isoforms in peptidoglycan remodelling, and hence chloroplast morphogenesis. Since cell morphogenesis is affected by variations in membrane fluidity^85^, investigation of the protein density dynamics in the chloroplast double membrane could also provide clues about the shape control for this organelle.

## Materials and Methods

### Plant Material and Growth Conditions

The “Gransden 2004” ecotype of the moss *Physcomitrella patens* (Hedw.) Bruch & Schimp. (IMSC accession number 40001) which was cultivated in bioreactors as described by Hohe and Reski^54^ was used in this study.

### RNA Isolation and Reverse Transcriptase–Polymerase Chain Reaction (RT-PCR)

Total RNA was extracted from *P. patens* protonema using TRIzol Reagent (Thermo Fisher Scientific Inc., Waltham, USA) according to the manufacturer’s protocol. 2 µg of total RNA was used for the first-strand cDNA synthesis using SuperscriptIII reverse transcriptase (Life Technologies, Carlsbad, CA, USA) according to the manufacturer’s protocol. This cDNA was used for the cloning of the coding sequence for PpFtsZ1-2 and PpFtsZ2-1.

### Molecular Cloning

#### Cloning of the reporter plasmid pAct5::Linker::EGFP-MAV4

Linker::EGFP^86^ was PCR-amplified from pJET::Linker::EGFP vector using the primers P1 and P2 (Supplementary Table S2), which introduced the restriction sites BglII and SacI at the 5′ and 3′ ends of the Linker::EGFP, respectively. The 5′ Linker located upstream of the EGFP encodes a flexible polyglycine sequence which is intended to prevent steric hindrance between EGFP and the protein it is fused to. The PCR-amplified Linker::EGFP was then cloned into the vector PpAct5::MAV4 (modified from Kircher *et al*.^87^) via BglII and SacI sites, generating the reporter plasmid PpAct5::Linker::EGFP-MAV4. This plasmid contains the promoter PpAct5, which is the endogenous promoter for the Actin5 gene in *P. patens*^88,89^ and a *NOS* terminator.

#### Cloning of the fusion constructs pAct5::PpFtsZ1-2::Linker::EGFP-MAV4 and pAct5::PpFtsZ2-1::Linker::EGFP-MAV4

The coding sequences for the PpFtsZ1-2 and PpFtsZ2-1 (without stop codons) were PCR-amplified from the cDNA using the primer pairs P3 and P4 for PpFtsZ1-2 and P5 and P6 for PpFtsZ2-1 (Supplementary Table S2), introducing the restriction sites SalI and BglII at the 5′ and 3′ ends of the coding sequence for PpFtsZ1-2 and KpnI and BglII at the 5′ and 3′ ends of the coding sequence for PpFtsZ2-1, respectively. These PCR products were then cloned into the reporter plasmid PpAct5::Linker::EGFP-MAV4 via the corresponding restriction sites, hence developing the fusion constructs PpAct5::PpFtsZ1-2::linker::EGFP and PpAct5::PpFtsZ2-1::linker::EGFP. These constructs were used for the transfection of moss protoplasts.

### Preparation of DNA for transfection

For acquisition of high amounts of plasmid DNA, the PureYield™ Plasmid Midiprep kit (Promega, Wisconsin, USA) was used according to the manufacturer’s protocol. The purified plasmid DNA was sterilised and concentrated based on the standard ethanol precipitation protocols^90^. Prior to transfection, 50 µg of the concentrated DNA was mixed with appropriate volumes of sterile 1 M Ca(NO_3_)_2_ and dH_2_O to attain a final concentration of 0.1 M Ca(NO_3_)_2_ in a final volume of 100 µl.

### Isolation and transfection of *P. patens* protoplasts

Moss protonema grown in bioreactor^54^ for 3 days were used for protoplast isolation and transfection based on the protocol described by Hohe *et al*.^91^. In a single transfection reaction the prepared 100 µl DNA sample (50 µg plasmid DNA in 0.1 M Ca(NO_3_)_2_) was added to 3 × 10^5^ protoplasts. The transfected protoplasts were incubated for 24 h in the dark. Subsequently, normal conditions (25 ± 1 °C; light-dark regime of 16:8 h light flux of 55 μmol s−1 m−2 from fluorescent tubes, Philips TL - 19 - 65 W/25) were applied until microscopy. The microscopic analysis was performed between the 4th and 7th day after transfection.

### Image acquisition and enhancement

The transfected cells were transferred into an Eppendorf tube and precipitated by gravity. The supernatant was discarded so that the cells were concentrated to a volume of 100 μl. 20 μl of these cells were directly transferred on a cover glass, which was subsequently laid on a glass slide. Edges of the cover glass were sealed with nail polish.

#### Confocal microscopy imaging of the knockout chloronema cells

All images were taken with a Leica TCS SP8 microscope (Leica Microsystems, Wetzlar, Germany) using HC PL APO CS2 63x/1.40 oil objective with a zoom factor of 2. The voxel size was 0.181 μm on the X-Y dimensions and 0.500 μm on the Z dimension. The pinhole was adjusted to 0.70 AU (66.8 µm). For the excitation WLL laser was applied at 488 nm with an intensity of 4%. The detection range for the chlorophyll autofluorescence was set to 664–725 nm.

#### Confocal microscopy imaging of the transfected protoplasts

All images were taken with a Leica TCS SP8 microscope (Leica Microsystems, Wetzlar, Germany) using HCX PL APO 100x/1.40 oil objective with a zoom factor of 10.6. The voxel size was 0.021 μm on the X-Y dimensions and 0.240 μm on the Z dimension. These voxel values were intended to exceed the optical resolution limit of the instrument, as the subsequent deconvolution process required oversampling of the image plane. The pinhole was adjusted to 0.70 AU (106.1 μm). For the excitation WLL laser was applied at 488 nm with an intensity of 4%. The detection range was set to 503–552 nm for the EGFP fluorescence and 664–725 nm for the chlorophyll autofluorescence. 40 of these images (n = 20 FtsZ1-2; n = 20 FtsZ2-1) were used for quantitative analysis.

#### Deconvolution and visualization

Deconvolution was performed using Huygens Professional version 17.04 (Scientific Volume Imaging, The Netherlands). The confocal image data sets of the FtsZ networks and the corresponding chloroplasts were initially deconvolved based on the Classical Maximum Likelihood Estimation (CMLE) algorithm using the theoretical point spread function of the Huygens software.

Maximum intensity projections (MIPs), blend renderings and isosurface renderings that were used for illustrations were all performed by using the IMARIS 9.1.0 software (Bitplane AG, Zurich, Switzerland). For illustration of the networks and chloroplasts, deconvolved image data were used. For illustration of the wild type and mutant chloronema tissue, non-deconvolved raw image data were used.

### Quantitative image analysis

A method to computationally quantify structural components and assembly of protein networks from 3D imaging previously developed and validated on data sets of FtsZ1-2^55,59,60^ was used in the present study. This methods allows for a quantitative assessment of the structural features of the network from i) a global perspective by analysing the shape of the network as a whole volume (shape descriptors) and ii) a local perspective by analysing the details of the structural components of the networks (element descriptors). Here, 40 confocal microscopy images (n = 20 images per isoform) were analysed with this method to identify and quantify structural differences in the phenotypes of the two isoforms. In the following the steps of the method are briefly described, whereas the extracted quantitative measures are explained in detail. For more information regarding the image processing steps we refer to Asgharzadeh *et al*.^55^.

First, the network is extracted from the deconvolved image data by means of a semi-automatic segmentation approach. Therefore, a local adaptive threshold algorithm is combined with a manual correction step (FEI Amira 6.2.0; Thermo Fisher Scientific, USA).

Second, the gross morphology of the network is studied as a whole. In this step a wrapped hull of the segmented network is calculated, which is defined as the summation of all convex hulls of the network in each stack of the image. This wrapped hull represents a solid outer surface representing the volume enclosing the network. Several shape descriptors are calculated based on combined analysis of the segmented network and its wrapped hull to quantitatively describe the overall morphology of the network. This includes: 1. The enclosed volume of the network, *V*_*EN*_, calculated as the volume of the outer surface. 2. The network volume, *V*_*PN*_, describing the volume of the segmented network. 3. The network volume density, *ρ*_*PN*_, representing the ratio of enclosed volume to network volume. Then a shape matrix is built, representing the covariance of outer surface created by the wrapped hull. Further shape descriptors calculated based on an analysis of the eigenvalues of the shape matrix are: 4. The greatest diameters of the network, ${d}_{PN}^{max}$, 5. The smallest diameters of the network, ${d}_{PN}^{min}$. 6. The stretch of the network, *St*_*PN*_∈[0,1], describing how stretched the shape of the network is. 7. The oblateness of the network, *Ob*_*PN*_∈[−1,1], describing how plate-like the network is. The calculation of the shape matrix and the shape descriptors are carried out by a set of in-house matlab codes (Matlab 2017a, MathWorks, USA).

Third, to analyse the details of the structural components of the FtsZ network a spatial graph consisting of points, nodes and segments is extracted. This extraction contains the following steps: 1. Detection of edges in the segmented image. 2. Calculating a distance map of all voxels from the nearest edge. 3. Finding the centreline of each filament based on this distance map. 4. Placing points at the centrelines in any position, where change in either the thickness or the direction of the filament occurs. This fragmentation of the network to its structural components allows for a quantitative assessment of the local structural features of the network. The following constituents represent the structural components of the network: 1. Points: Placed at filaments where a local change in segment characteristic such as orientation or thickness occurs. 2. Elements: Structural component connection points. 3. Nodes: Points that are connected to more than two other points. Nodes are meeting points of the filaments in the network. 4. Segments: The filaments connecting one node to another. 5. Connection: Joints of the network resulting from filaments meeting in a node. Moreover, a distance map of the segmented image is calculated which evaluates the Euclidean distance of each voxel to the closest zero valued voxel (edge of the filaments). This distance map is utilized to calculate the thickness of the filamentous structure at each point of the constructed spatial graph (FEI Amira 6.2.0; Thermo Fisher Scientific, USA).

Fourth, a quantitative description of the local structural features of the networks is achieved by calculating the following element descriptors: 1. Number of nodes in the network, *N*_*n*_. 2. Node thickness, *th*_*n*_. 3. Node density, *ρ*_*n*_, describing the number of nodes per volume unit in the network. It has to be kept in mind that this is not the same as the network volume density. 4. Node-to-node distance, *d*_*nn*_, describing the Euclidean distance between two neighbouring nodes. 5. Node-to-surface distance, *d*_*ns*_, representing the distance of a node to the outer surface. 6. Node-to-centre distance, *d*_*nc*_, describing the distance of the nodes to the center of gravity of the network. 7. Node-to-surface to node-to-centre distance ratio. 8. Compactness, *C*_*PN*_∈[0,1], defined as:

$${C}_{PN}=\frac{{d}_{nc}-{d}_{ns}}{{d}_{nc}},$$

representing how densely the nodes are placed in the network. 9. Node-to-surface to node-to-center ratio, showing weather nodes are placed closer to the surface or the center of gravity of the network. 10. The total number of segments, *N*_*s*_. 11. Segment length, *L*_*s*_, describing the length of individual filaments between nodes. 12. Segment curvature, *κ*_*s*_, calculated as the Menger curvature of the segments. 13. Mean segment thickness, *th*_*s*_, as the mean value of the thickness calculated at all points on a segment. 14. Segment inhomogeneity, *I*_*s*_, describing how often local changes in thickness or orientation occur in a segment. 15. Mean point-to-point distance, *d*_*pp*_, representing the distance between neighboring points on a segment. 16. The mean number of connections per node, *n*_*c*_, describing number of segments meeting in one node. 17. Open nodes, *n*_*oe*_, calculated as the percentage of all the nodes which are not connected to more than one other node. 18. The mean angles between segments in a connection, *θ*_*c*_. Calculation of these element descriptors is performed by a set of in-house matlab codes (Matlab 2017a, MathWorks, USA).

### Statistical analysis

For all outcome measures, differences between isoforms were assessed by unpaired Student’s t-test. All values are presented as mean ± standard deviation. Statistical significance was set at p = 0.05. Distributions are calculated as mean ± standard deviation of normalised probability or normalised cumulative probability of descriptors from the network of each image.

## Electronic supplementary material

Supplementary Information

Supplementary Video S1

Supplementary Video S2
