# Supplementary material for: Cytological analysis and structural quantification of FtsZ1-2 and FtsZ2-1 network characteristics in Physcomitrella patens
Source: Sci Rep. 2018 Jul 24;8:11165. doi: 10.1038/s41598-018-29284-y (PMC6057934; doi:10.1038/s41598-018-29284-y)
Supplement: Supplementary file 1 — Supplementary Information [file 41598_2018_29284_MOESM1_ESM.docx]

***Supplementary Information***

**Cytological analysis and structural quantification of FtsZ1-2 and Ftsz2-1 network characteristics in *Physcomitrella patens***

Bugra Özdemir^1,☯^, Pouyan Asgharzadeh^2,4,☯^, Annette I. Birkhold^2^, Stefanie J. Mueller^3^, Oliver Röhrle^2,4*^, Ralf Reski^1,5,6*^

^1^ Plant Biotechnology, Faculty of Biology, University of Freiburg, Schaenzlestr. 1, 79104 Freiburg, Germany

^2^ Institute of Applied Mechanics, University of Stuttgart, Pfaffenwaldring 7, 70569 Stuttgart, Germany

^3^ INRES – Chemical Signalling, University of Bonn, Friedrich-Ebert-Allee 144, 53113 Bonn, Germany

^4^ Stuttgart Center for Simulation Science (SimTech ), University of Stuttgart, Pfaffenwaldring 5a, 70569 Stuttgart, Germany

^5^ BIOSS – Centre for Biological Signalling Research, University of Freiburg, Schaenzlestr. 18, 79104 Freiburg, Germany

^6^ Freiburg Center for Interactive Materials and Bioinspired Technologies (FIT), University of Freiburg, Georges-Köhler-Allee 105, 79110 Freiburg, Germany

* Corresponding authors: [roehrle@simtech.uni-stuttgart.de](mailto:roehrle@simtech.uni-stuttgart.de) & [ralf.reski@biologie.uni-freiburg.de](mailto:ralf.reski@biologie.uni-freiburg.de)

^☯^ These authors contributed equally to this work.

ORCID IDs: 0000-0002-1934-6525 (O.R.), 0000-0002-5496-6711 (R.R.)

Supplementary Table S1: List of the proteins searched *in silico* against the *P. patens* genome to find putative homologs

| **Protein** | **Species** | **Strain** | **NCBI ID** | **Relevant publications** |
| --- | --- | --- | --- | --- |
| MreB | *Nostoc sp.* | PCC 7120 | BAB77611.1 | Hu *et al*. 2007^1^ |
|  | *Synechococcus elongatus* | PCC7942 | ABB56332.1 | Savage *et al*. 2010^2^ |
|  | *Caulobacter crescentus* | CB15N | NP_420354.1 | Harris *et al*. 2014^3^, Dye *et al*. 2011^4^ |
|  | *Bacillus subtilis* | 168 | NP_390681.2 | Jones *et al*. 2001^5^ |
|  | *Escherichia coli* | MG1655 | NP_417717 | Ouzounov *et al*. 2016^6^ |
| RodZ | *Escherichia coli* | MG1655 | NP_417011 | Shiomi *et al*. 2008^7^ |
|  | *Escherichia coli* | BW25113 | AIN32917.1 | Shiomi *et al*. 2008^7^, Shiomi *et al*. 2013^8^ |
| CreS | *Caulobacter crescentus* | CB15N | DAA05203.2 | Ausmees *et al*. 2003^9^ |
| FilP | *Streptomyces coelicolor* | A3(2) | NP_629535.1 | Bagchi *et al*. 2008^10^ |


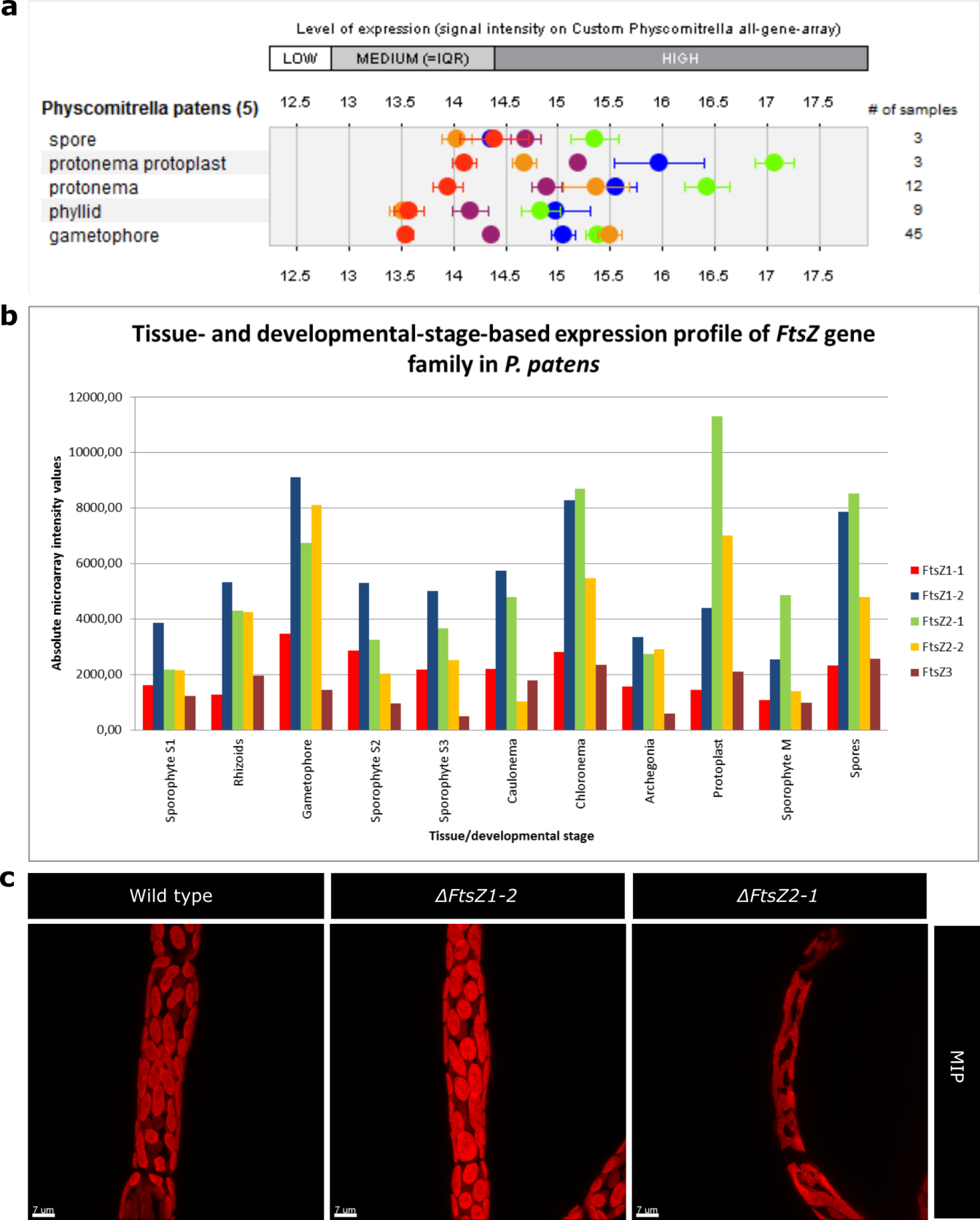


Supplementary Figure S1. a-b: Comparison of expression profiles of all members of the *FtsZ* gene family based on their absolute microarray intensity values corresponding to various tissue types and growth stages of *P. patens*. Colour code: *FtsZ1-1*: red, *FtsZ1-2*: blue, *FtsZ2-1*: green, *FtsZ2-2*: orange, *FtsZ3*: magenta. a: The graph was generated by using the publicly accessible database Genevestigator V3^11,12^. In general, *FtsZ1-2* and *FtsZ2-1* show higher expression than the other three homologs, in particular in protoplasts, protonema and leaves (phyllids). b: The chart was generated based on the publicly accessible gene expression data, which was retrieved from the *Physcomitrella* eFP Browser^13^ at <http://bar.utoronto.ca/efp_physcomitrella/cgi-bin/efpWeb.cgi>. According to this analysis, the expressions of *FtsZ1-2* and *FtsZ2-1* are higher than that of the other homologs in the majority of the tissues. Namely, all stages of sporophyte, caulonema, chloronema and spores show higher expression levels for these two homologs compared to the other members of the family. *FtsZ1-2* expression reaches a peak in gametophores, while the maximum expression of *FtsZ2-1* occurs in protoplasts. c: Comparison of the morphology of chloroplasts in the chloronema cells of wild type, Δ*ftsZ1-2* and Δ*ftsZ2-1*. Chloroplasts of wild type cells have roughly a roundish shape, except that the chloroplasts that are in the process of division become elongated. Compared to the chloroplasts of the wild type cells, the chloroplasts of Δ*ftsZ1-2* cells show no detectable morphological differences, whereas the chloroplasts of the Δ*ftsZ2-1* cells show complete loss of chloroplast integrity. The confocal image data were acquired based on the chlorophyll autofluorescence and rendered by maximum intensity projection (MIP) for illustration using the IMARIS software (Bitplane).


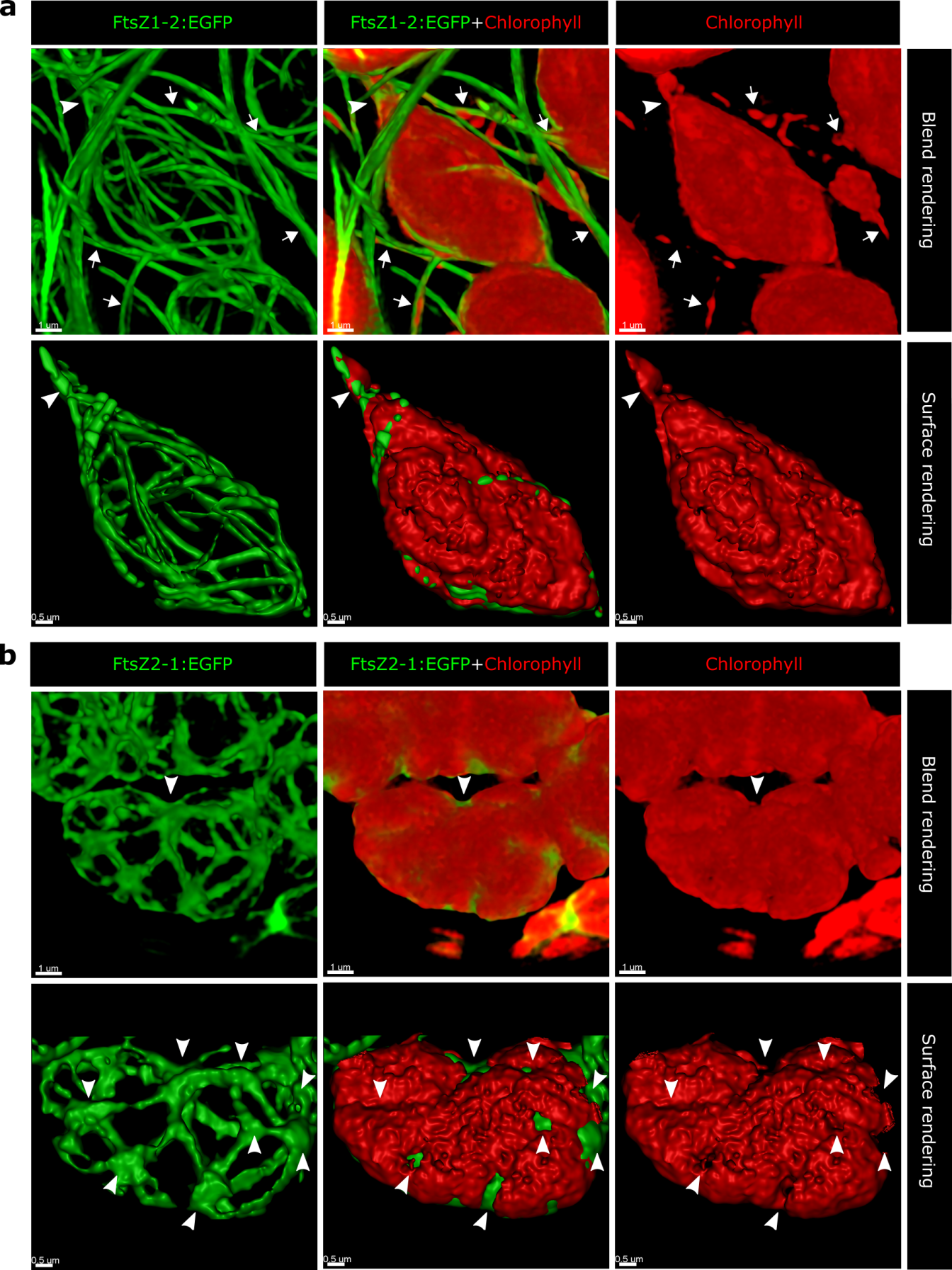


Supplementary Figure S2. Further examples of morphological patterns of chloroplasts carrying FtsZ1-2::EGFP (a) and those carrying FtsZ2-1::EGFP (b). Confocal image z-stacks were deconvolved via Huygens software (Scientific Volume Imaging). Blend rendering and surface rendering were performed on selected confocal image data for each isoform by using IMARIS software (Bitplane). Surface rendering was performed by choosing a single chloroplast in the middle of the image as the object and excluding the surrounding chloroplasts and networks during segmentation. (a) Volume reconstruction (blend rendering) of the image data shows the deformation of chloroplast morphology in the presence of FtsZ1-2::EGFP. These consist of tapering of poles (arrowheads in the chlorophyll channel) and tubular protrusions from chloroplast surface (arrows in the chlorophyll channel). Surface rendering of the image data shows that the pointed tip of the chloroplast border is aligned with a thick bundle of the FtsZ1-2::EGFP filaments (arrowheads). (b) 3D reconstructions of FtsZ2-1::EGFP network and the corresponding chloroplast (blend rendering) show that the chloroplast shape is irregular and a meganode is located at the position of indentation (arrowhead). Surface rendering reveals several more indentations coincident with meganodes at the corresponding positions (arrowheads).

**Supplementary Video S1**. Volume and surface renderings of an FtsZ1-2::EGFP network and the chloroplast carrying the network created by using IMARIS software (Bitplane) and shown at different angles. The surface renderings were performed after isolating a single chloroplast and network by means of the “splitting objects” tool of IMARIS software (Bitplane). Typical morphological patterns of the chloroplasts carrying exogenous FtsZ1-2 consist of sharp corners, tapered poles and tubules protruding from chloroplast surface, accompanied by FtsZ1-2 filaments which co-align with these tubules.

**Supplementary Video S2**. Volume and surface renderings of an FtsZ2-1::EGFP network and the chloroplast carrying the network created by using IMARIS software (Bitplane) and shown at different angles. The surface renderings were performed after isolating a single chloroplast and network by means of the “splitting objects” tool of IMARIS software (Bitplane). Typical morphological patterns of the chloroplasts carrying exogenous FtsZ2-1 consist of surface indentations which ocur concomitantly with meganodes falling within the indented sites.

Supplementary Table S2: List of the primers used for cloning of the constructs

| **Primer Name** | **Sequence** |
| --- | --- |
| P1 | TTTAGATCTGGAGGTGGAGGTGGAGCT |
| P2 | TTTGAGCTCCTACTTGTACAGCTCGTCCAT |
| P3 | AGGTCGACATGGGCTCTGTCGCGGT |
| P4 | CCAGATCTCAAAAACCCCTTTCGGTTAAGAC |
| P5 | ACGGTACCATGGCGTTGTTTAGTGG |
| P6 | CCAGATCTATGACGTGTCTGGCCTC |

**References**

1. Hu, B., Yang, G., Zhao, W., Zhang, Y. & Zhao, J. MreB is important for cell shape but not for chromosome segregation of the filamentous cyanobacterium *Anabaena sp.* PCC 7120. *Molecular Microbiology* **63**, 1640–1652 (2007).
2. Savage, D. F., Afonso, B., Chen, A. H. & Silver, P. A. Spatially ordered dynamics of the bacterial carbon fixation machinery. *Science* **327**, 1258–1261 (2010).
3. Harris, L. K., Dye, N. A. & Theriot, J. A. A *Caulobacter* MreB mutant with irregular cell shape exhibits compensatory widening to maintain a preferred surface area to volume ratio. *Molecular Microbiology* **94**, 988–1005 (2014).
4. Dye, N. A., Pincus, Z., Fisher, I. C., Shapiro, L. & Theriot, J. A. Mutations in the nucleotide binding pocket of MreB can alter cell curvature and polar morphology in *Caulobacter*. *Molecular Microbiology* **81**, 368–394 (2011).
5. Jones, L. J., Carballido-López, R. & Errington J. Control of cell shape in bacteria: helical, actin-like filaments in Bacillus subtilis. *Cell* **104,** 913-922 (2001)
6. Ouzounov, N. *et al.* MreB Orientation correlates with cell diameter in *Escherichia* *coli*. *Biophysical* *Journal* **111**, 1035–1043 (2016).
7. Shiomi, D., Sakai, M. & Niki, H. Determination of bacterial rod shape by a novel cytoskeletal membrane protein. *EMBO Journal* **27**, 3081–3091 (2008).
8. Shiomi, D. *et al.* Mutations in cell elongation genes mreB, mrdA and mrdB suppress the shape defect of RodZ-deficient cells. *Molecular* *Microbiology* **87**, 1029–1044 (2013).
9. Ausmees, N., Kuhn, J. R. & Jacobs-Wagner, C. The bacterial cytoskeleton: an intermediate filament-like function in cell shape. *Cell* **115**, 705-713 (2003).
10. Bagchi, S., Tomenius, H., Belova, L. M. & Ausmees, N. Intermediate filament-like proteins in bacteria and a cytoskeletal function in Streptomyces. *Molecular Microbiology* **70**, 1037-1050 (2008).
11. Hruz, T., *et al*. Genevestigator V3: A reference expression database for the meta-analysis of transcriptomes. *Advances in Bioinformatics* **2008**, 1–5 (2008).
12. Hiss, M., *et al*. Large-scale gene expression profiling data for the model moss *Physcomitrella patens* aid understanding of developmental progression, culture and stress conditions. *Plant Journal* **79**, 530-539 (2014).
13. Ortiz-Ramírez, C., *et al.* A transcriptome atlas of *Physcomitrella* *patens* provides insights into the evolution and development of land plants. *Molecular Plant* **9**, 205–220 (2016).
